# Phenotypic and Genotypic Characteristics of a Tigecycline-Resistant *Acinetobacter pittii* Isolate Carrying *bla*_NDM–1_ and the Novel *bla*_OXA_ Allelic Variant *bla*_OXA–1045_

**DOI:** 10.3389/fmicb.2022.868152

**Published:** 2022-05-04

**Authors:** Zixuan Ding, Zhaoyinqian Li, Yuanqing Zhao, Jingchen Hao, Tingting Li, Yao Liu, Zhangrui Zeng, Jinbo Liu

**Affiliations:** Department of Laboratory Medicine, The Affiliated Hospital of Southwest Medical University, Luzhou, China

**Keywords:** *Acinetobacter pittii*, tigecycline resistance, carbapenem resistance, OXA-1045, whole genome sequencing

## Abstract

A tigecycline-resistant *Acinetobacter pittii* clinical strain from pleural fluid carrying a *bla*_NDM–1_ gene and a novel *bla*_OXA_ gene, *bla*_OXA–1045_, was isolated and characterized. The AP2044 strain acquired two copies of the *bla*_NDM–1_ gene and six antibiotic resistance genes (ARGs) from other pathogens. According to the whole-genome investigation, the GC ratios of ARGs (50–60%) were greater than those of the chromosomal backbone (39.46%), indicating that ARGs were horizontally transferred. OXA-1045 belonged to the OXA-213 subfamily and the amino acid sequence of OXA-1045 showed 89% similarity to the amino acid sequences of OXA-213. Then, *bla*_OXA–1045_ and *bla*_OXA–213_ were cloned and the minimum inhibitory concentrations (MICs) of β-lactams in the transformants were determined using the broth microdilution method. OXA-1045 was able to confer a reduced susceptibility to piperacillin and piperacillin-tazobactam compared to OXA-213. AP2044 strain exhibited low pathogenicity in *Galleria mellonella* infection models. The observation of condensed biofilm using the crystal violet staining method and scanning electron microscopy (SEM) suggested that the AP2044 strain was a weak biofilm producer. Quantitative reverse transcription-PCR (qRT-PCR) was used to detect the expression of resistance-nodulation-cell division (RND) efflux pump-related genes. The transcription level of *adeB* and *adeJ* genes increased significantly and was correlated with tigecycline resistance. Therefore, our genomic and phenotypic investigations revealed that the AP2044 strain had significant genome plasticity and natural transformation potential, and the emergence of antibiotic resistance in these unusual bacteria should be a concern for future investigations.

## Introduction

*Acinetobacter* spp. has been a global threat in the healthcare setting since they rapidly develop resistance to antibiotics. Among these species, *Acinetobacter baumannii*, *Acinetobacter nosocomialis*, and *Acinetobacter pittii* are the most common isolates in hospitals and are associated with nosocomial infections (Weber et al., 2015; Almasaudi, 2018). *A. pittii*, previously known as *Acinetobacter* genomic species three, is the most frequently isolated from nosocomial infections among inpatients in general wards and intensive care units (ICU) in Germany and is increasingly found in France, South Asia, and even China (Yang et al., 2012; Schleicher et al., 2013; Ji et al., 2014; Jones et al., 2015; Al Atrouni et al., 2016; Singkham-In and Chatsuwan, 2018).

The emergence of carbapenem-resistant bacteria has been a significant challenge to clinicians worldwide with limited therapeutic options. Tigecycline is a member of the glycylcyclines and serves as a last resort to treat multidrug-resistant (including carbapenem-resistant) *Acinetobacter* infections (Yang et al., 2017). The resistance mechanism of *Acinetobacter* spp. to carbapenem antibiotics is based on the production of the carbapenem-hydrolyzing class D β-lactamases (CHDLs), such as *bla*_OXA–23–like_ (Singkham-In and Chatsuwan, 2018), *bla*_OXA–24–like_ (Ji et al., 2014), *bla*_OXA–58–like_ (Ji et al., 2014), *bla*_OXA–72–like_ (Montealegre et al., 2012), and other variants. They can be intrinsic and have a limited ability to hydrolyze carbapenems, but can also result in a resistant phenotype, particularly when overproduced (Tietgen et al., 2021). There are three characterized resistance-nodulation-cell division (RND) efflux pumps: AdeABC, AdeFGH, and AdeIJK. They have been linked to antibiotic resistance, especially tigecycline resistance. Among them, the AdeABC and AdeIJK efflux pumps have been shown to play a major role in tigecycline resistance (Ruzin et al., 2007; Leus et al., 2018). In contrast to intrinsic determinants, acquired antibiotic resistance, such as New Delhi metallo-β-lactamase (NDM), has recently gained importance and contributed to reduced susceptibility of the *Acinetobacter* species. Specifically, the emergence of NDM-1-producing *A. pittii* was first reported in China, (Yang et al., 2012), followed by other regions, such as Korea (Sung et al., 2015), France (Pailhoriès et al., 2017), and Denmark (Hammerum et al., 2015) before spreading around the world.

The objectives of the present study were to systematically analyze the tigecycline-resistant *A. pittii* isolated from an ICU patient, which co-produces *bla*_*NDM–1*_ and *bla*_OXA–1045_. As a result, a novel OXA enzyme was found and its active spectrum was identified. The strain was also described using an antimicrobial susceptibility profile, biofilm-forming ability, *Galleria mellonella* infection model, expression of efflux pump-related genes, conjunction experiment, and whole-sequence analysis.

## Materials and Methods

### Data Collection, Bacterial Isolate, and Susceptibility Testing

A total of 104 non-duplicate *Acinetobacter* spp. isolates were obtained between January 2020 and April 2021 in the Affiliated Hospital of Southwest Medical University. Bacterial identification was confirmed by matrix-assist laser desorption ionization time-of-flight mass spectrometry (MALDI-TOF MS) (Bruker, Bremen, Germany) and 16s rRNA sequencing (Supplementary Datasheet 1). The antimicrobial susceptibility profiles were tested by MicroScan Walk-Away 96 Plus system (Siemens, Germany). The minimum inhibitory concentrations (MICs) of meropenem, imipenem, tigecycline, and colistin were determined using the broth microdilution method, and results were interpreted following the Clinical and Laboratory Standards Institute 2020 standards (CLSI, 2020). *Escherichia coli* ATCC25922 and *Pseudomonas aeruginosa* ATCC27853 were used as a quality control. ATCC19606 (lab-WT) was used as the reference strain in this study.

### Whole-Genome Sequencing and Sequence Analysis

Genomic DNA of *Acinetobacter pittii* AP2044 strain was extracted using a DNA extraction kit (Qiagen, Hilden, Germany) and then sequenced using the Illumina NovaSeq 6000 PE150 (Illumina Inc, San Diego, CA, United States) and nanopore platforms (Sangon Biotech, Shanghai, China). Read sequences were *de novo* assembled using Canu workflow (v1.7) (Koren et al., 2017). Prokka (v1.10) was utilized to predict coding genes, tRNA, and rRNA in the assembled genome Seemann, 2014. All genomic data were uploaded in the National Center for Biotechnology Information (NCBI) database under the accession number CP087716-CP087718. A novel OXA variant was then identified and termed OXA-1045 (accession no. OL790815). The ResFinder^1^ and NCBI BLAST^2^ were used to determine the acquired resistance genes. The GC Content Calculator web tool^3^ was used to measure the GC ratio. Additionally, the IS finder^4^ and VFDB^5^ databases were used to determine the insertion sequence (IS) elements and virulence genes. The genomic island (GI) sequences were predicted based on GI prediction software packages (IslandPATH-DIMOB) (Hsiao et al., 2003). Chromosome comparison was performed using BLAST Ring Image Generator (BRIG) in the default settings (Alikhan et al., 2011).

### Conjugation

As previously mentioned, mating experiments were conducted in broth and on filters using *Escherichia coli* J53 AizR (an azide resistant strain of J53) as the recipient at 37°C (Fu et al., 2012). Potential transconjugants were selected on Luria-Bertani (LB) broth agar plates containing 0.5 mg/L of imipenem and 180 mg/L of sodium azide (Xiang et al., 2020). Transconjugants were verified by MALDI-TOF MS and polymerase chain reaction (PCR), respectively.

### Characterization of the New β-Lactamase OXA-1045

Sequence alignment of OXA-1045 with OXA-213 was investigated using Clustal Omega (Sievers et al., 2011) and ESPript 3.0^6^. The secondary structure of OXA-1045 β-lactamase was predicted using the JPred4 online tool^7^, which is based on neural networks. To investigate the phylogenetic relationship of OXA-213-like proteins, we collected a total of 78 OXA amino acid sequences from Beta-Lactamase DataBase (BLDB)^8^ (accessed 11 March 2022). Protein sequence alignment of these aa sequences and of OXA-1045 was computed using Clustal Omega. This alignment was used to reconstruct the maximum-likelihood phylogeny by MEGA 7.0 (Kumar et al., 2016).

### Cloning of β-Lactamase Genes and Expression

To evaluate the impact of resistance-determinant genes on MICs, *bla*_OXA–1045_ and *bla*_OXA–213_ were cloned into the vector pET28b (MiaoLingBio, Wuhan, China). To make pET28b-OXA1045 and pET28b-OXA213, PCR amplification and vector pET-28b were digested with *Bam*HI and *Xho*I and then ligated to the pET-28b vector (Invitrogen, Carlsbad, California, United States). As previously described, the generated plasmid was chemically converted into *E. coli* strain BL21 (Sigma-Aldrich, St. Louis, MO, United States) (Liu et al., 2021). Potential transformants containing pET28b-OXA1045 were identified on LB agar plates (Sigma-Aldrich, St. Louis, MO, United States) containing 20 mg/L of kanamycin (TransGen, Beijing, China). PCR primers PET28AVF2/PET-VF were used to screen colonies on plates, followed by Sanger sequencing (Supplementary Datasheet 1). The empty vector pET-28b was turned into BL21 for use as a control.

MICs of ampicillin, ampicillin-sulbactam, piperacillin, piperacillin-tazobactam, oxacillin, cefazolin, cefoxitin, cefuroxime, ceftazidime, cefotaxime, imipenem, and meropenem for the transformants containing pET28b-OXA1045 (BL21:pET28b-OXA1045) and pET28b-OXA213 (BL21:pET28b-OXA213) were determined by the broth microdilution method. MICs for ampicillin in the presence of 4 mg/L of sulbactam were also determined based on the methods used to establish MICs for piperacillin-tazobactam.

### Biofilm Formation Assay

The biofilm formation ability of AP2044 strain and lab-WT was determined by the crystal violet staining method and field-emission scanning electron microscope (FE-SEM) as described previously with minor adjustment. Briefly, the strains were cultured overnight and adjusted to a final OD_600_ of 0.1. A total of 20 μL of each bacterial suspension and 180 μL of LB broth (Haibo, Qingdao, China) were inoculated into a 96-well polystyrene microtiter plate (Costar#3524, Corning, United States) in triplicate. After incubation at 37°C overnight, the plate was washed with phosphate-buffered saline (PBS, Solarbio, Beijing, China) to remove planktonic cells, and the plate was stained with crystal violet (Solarbio, Beijing, China) and solubilized with 95% ethanol (v/v), after which its absorbance was measured at 570 nm.

In addition, the biofilm formation capacity of each strain was used for SEM analysis. Biofilms were cultured for 24 h on coverslips as described above and planktonic cells were removed using PBS. First, the cells were fixed with 2.5% glutaraldehyde in PBS for 4 h. Second, the cells were gently washed with PBS twice. The samples were then gradually treated with ethanol (30, 50, 70, 80, 90, and 100%) for 20 min at each concentration. The dried samples were coated with platinum and visualized using FE-SEM (Thermo Fisher Scientific, Waltham, Massachusetts, United States).

### *Galleria mellonella* Infection Model

Prior to all injections, *Galleria mellonella* larvae were stored at 20°C for 4 h to increase susceptibility to infection. A total of 10 μL of bacterial suspension of AP2044 strain and lab-WT were injected into the last left proleg of *G. mellonella* larvae and incubated at 37°C. Larvae were scored for survival every 24 h for 7 days of incubation. Then, 15 larvae were injected at a concentration of 10^6^CFU/mL of each strain, which was repeated three times. A negative control group (10 μL of PBS buffer were injected) was set for each batch of experiments. Larvae were identified as dead when failure to move in response to external touch was noted (Kim et al., 2021).

### RNA Isolation and qPCR

Total RNA extraction and reverse transcription were conducted according to a previously described protocol (Tang et al., 2020). The quantitative reverse transcription-PCR (qRT-PCR) was used to detect the expression level of efflux pump-related genes (Supplementary Datasheet 1). The 2 ^–ΔΔCt^ method was used to calculate the fold change of mRNA expression. The relative gene expression level was compared to the control sample (tigecycline-susceptible *A. pittii*, TSAP) (Salehi et al., 2021), which was assigned a value of 1 arbitrary unit. All assays were performed in triplicate in three independent cultures.

## Results and Discussion

### Bacterial Isolate

A total of 69 carbapenem-resistant *Acinetobacter* spp. samples were obtained from sputum (40, 58%), urine (13, 18.8%), secreta (6, 8.7%), pleural fluid (6, 8.7%), and blood (4, 5.8%). The majority of them (35, 50.7%) was collected from the ICU. Among these clinical isolates, 36 (52.2%) were considered as extensively drug-resistant (XDR, the isolates are resistant to all antimicrobial classes, except colistin and/or tigecycline). Surprisingly, only the AP2044 strain was resistant to tigecycline. The MICs of the AP2044 strain increased drastically compared to the MICs of ATCC19606 among all tested antibiotics (Table 1). The increases were eightfold for gentamicin, 16-fold for piperacillin, more than 32-fold for ciprofloxacin, 64-fold for tigecycline more than 256-fold for carbapenems, more than 64-fold for tetracycline, and in the range more than 32–256-fold for cephalosporins. However, MICs of colistin were almost the same between the two strains, which meant that they are all susceptible to colistin. The AP2044 strain was recovered from a patient with a lung tumor who underwent multiple surgical and invasive procedures at the ICU. With previous treatments of ceftazidime, sulperazon, and moxifloxacin, the patient was polymicrobial-positive, which included *Pseudomonas aeruginosa, A. pittii*, and *Stenotrophomonas maltophilia.*

**TABLE 1:** MICs (mg/L) of common antibiotics in AP2044 and ATCC19606.

	AP2044	ATCC19606
Gentamicin	256	32
Meropenem	256	<1
Imipenem	256	<1
Ciprofloxacin	32	<1
Ceftazidime	>1,024	4
Ceftriaxone	>1,024	32
Cefotaxime	>1,024	16
Cefepime	>1,024	16
Tetracycline	>128	2
Tigecycline	64	1
Piperacillin	512	32
Colistin	<0.5	<0.5

### Conjugation and Whole-Genome Analysis of AP2044

**FIGURE 1:**
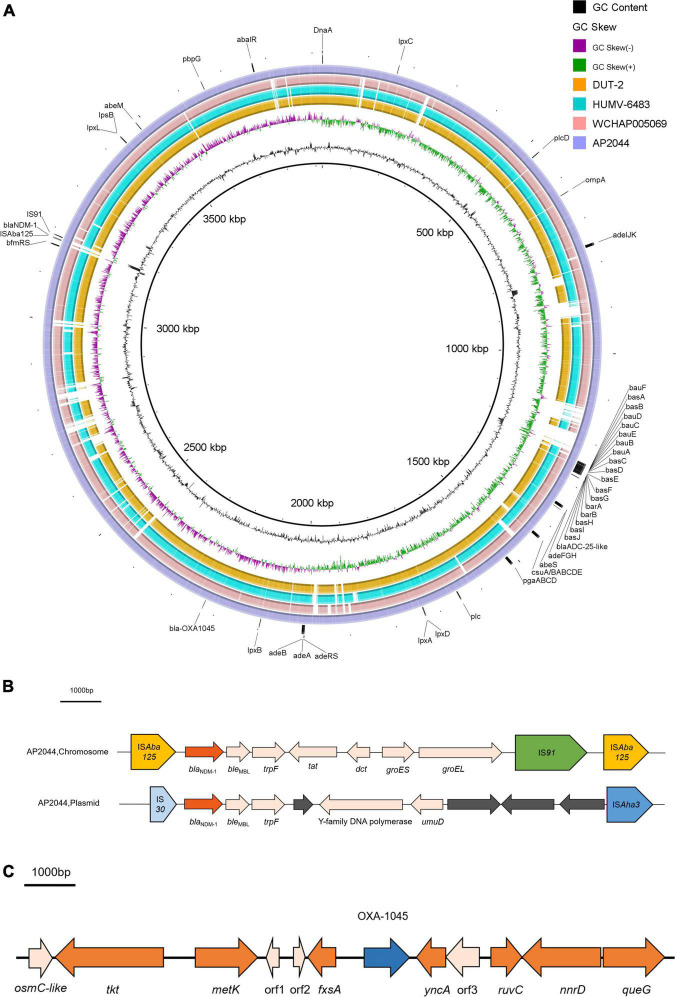
**(A)** Chromosomal genomic sequence of *A. pittii* AP2044 strain. Alignment of AP2044 with DUT2, HUMV-6483, and WCHAP005069. AP2044 is the closest to DUT2 (accession no. CP014651.1) and HUMV-6483 (accession no. CP021428.1). HUMV-6483 was recovered in a hospital of a neighboring city (Chengdu, China) in 2018, with an 89% coverage and 96.47% identity. Annotations are provided by ResFinder^1^, IS Finder^4^, and VFDB^5^ analysis. **(B)** Genetic environment of *bla*_NDM–1_ located on chromosome and pAP2044-1 from AP2044 strain. **(C)** Genetic environment of *bla*_OXA–1045_ from AP2044 strain.

The genome of AP2044 was assembled into three contigs of 4,248,736 bp, consisting of one chromosomal backbone and two plasmids. The chromosome, pAP2044-1, and pAP2044-2 had 3,921,810, 283,349, and 43,577 bp and 39, 39.4, and 39.14% G + C content, respectively (Table 2). The genome had 18 rRNA operons, 74 tRNAs, and 4,128 predicted protein coding sequences. Indeed, conjugation experiments of the AP2044 strain failed to transfer the plasmid into an *E. coli* recipient. Antimicrobial resistance can be acquired through horizontal gene transfer (HGT) of antibiotic resistance genes (ARGs) and plasmids are critical for HGT and serve as a support for other mobile genetic elements (MGEs) (Vrancianu et al., 2020). The plasmid carrying *bla*_NDM–1_, pAP2044-1, is similar to plasmid pXBB1-9 (accession no. CP010351.1) from *Acinetobacter johnsonii* and pALWED1.1 (accession no. KX426227.1) from *Acinetobacter lwoffii*. pAP2044-2 plasmid shows similarity among the sequenced plasmids to p1_010059 from *Acinetobacter junii* (accession no. CP028798.2) and pACI-235c from *Acinetobacter* sp. (accession no. CP026414.1). We speculated that pAP2044-1 and pAP2044-2 plasmids have narrow bacterial host spectrums, which may have a dominant transmission in *Acinetobacter* spp. The genome analyses demonstrated that the AP2044 strain possessed a total of 12 ARGs linked to five different classes of antibiotics, including carbapenem, β-lactam, aminoglycoside, macrolide, and sulfonamide (Figure 1A) (Table 3). The GC content of a gene can be compared to the whole genome of an organism to determine if that gene comes from that organism. If this is the case, the two GC content profiles are likely to be the same (Evans and Amyes, 2014). Furthermore, the GC ratios (50–60%) of several ARGs, including *bla*_NDM–1_, *aac(3″)-IIb, aph(3″)-Ib, aph(6)-Id*, and *sul2*, were much greater than those of the chromosomal backbone (39.46%), implying that the ARGs were acquired from other bacterial species. Therefore, we used the Center for Genomic Epidemiology database^9^ to examine the homology of the ARGs to see if they originated from other bacterial species. Eight of the 12 ARGs appeared to be originating from other pathogens, with gene homologies exceeding 99% identities (Table 3).

**TABLE 2:** Genome characteristics of *Acinetobacter pittii* strain AP2044.

Context	Size (bp)	G + C (%)	No. of predicted ORFs
Chromosome	3,921,810	39.0	3,795
Plasmid pAP2044-1	283,349	39.4	290
Plasmid pAP2044-2	43,577	39.1	47

**TABLE 3:** Resistance gene distribution in *Acinetobacter pittii* strain AP2044.

	Resistance gene	Identity%	Query/template length	Position in context	Predicted phenotype	Source	GC content of AR gene (%)	Accession number
Chromosome	*bla*_NDM–1_	100	813/813	3178789.3179601	Beta-lactam resistance	*Klebsiella pneumoniae* plasmid pKpANDM-1	61	FN396876
	*bla*_ADC–25_	91.93	1,152/1,152	1279039.1280190	Beta-lactam resistance	*Acinetobacter baumannii* strain	34	EF016355
	*bla*_OXA–1045_	100	822/822	2227748.2228569	Beta-lactam resistance	*Acinetobacter pittii strain* AP2044	36	OL790815
pAP2044-1	*aac(3″)-IIb*	99.88	861/861	24804.25664	Aminoglycoside resistance	*Escherichia coli*	58	EU022314
	*aph(3′)-Via*	100	780/780	201505.202284	Aminoglycoside resistance	*Acinetobacter baumannii*	32	X07753
	*bla*_NDM–1_	100	813/813	194810.195622	Beta-lactam resistance	*Klebsiella pneumoniae* plasmid pKpANDM-1	61	FN396876
pAP2044-2	*aph(3″)-Ib*	100	804/804	22734.23537	Aminoglycoside resistance	*Shigella flexneri* plasmid Pstr1	55	AF321551
	*aph(6)-Id*	100	837/837	23537.24373	Aminoglycoside resistance	*Escherichia coli* plasmid RSF1010	55	M28829
	*mph(E)*	100	885/885	6420.7304	Macrolide resistance	Uncultured bacterium plasmid pRSB105	36	DQ839391
	*msr(E)*	100	1,476/1,476	4889.6364	Macrolide, Lincosamide and Streptogramin B resistance	*Pasteurella multocida*	39	FR751518
	*sul2*	100	816/816	20562.21377	Sulfonamide resistance	*Vibrio cholerae*	60	AY034138
	*tet(39)*	99.91	1,122/1,122	8331.9452	Tetracycline resistance	*Acinetobacter baumannii* strain RCH52 plasmid pRCH52-1	40	KT346360

Bacterial survival tactics against aminoglycoside antibiotics include altering enzymes to inactivate aminoglycosides, increasing efflux pump production, lowering membrane permeability, and interfering with aminoglycoside binding *via* modification of 16S ribosomal RNA (Su et al., 2018). Interestingly, the AP2044 genome has four distinct aminoglycoside resistance genes, conferring remarkable cell resistance to a wide variety of aminoglycoside antibiotics (eightfold greater than ATCC19606, Table 1, and Supplementary Datasheet 2). Two genes [*aac(3″)-IIb* and *aph(6)-Id*] were most likely transmitted from *E. coli*, whereas one *aph(3″)-Ib* gene was likely transferred from *Shigella flexneri*. This might add phosphate, acetyl, adenyl, or methyl groups to aminoglycosides to alter them. Macrolide antibiotics inhibit protein synthesis by targeting the bacterial ribosome (Vázquez-Laslop and Mankin, 2018). Furthermore, the *msr(E)* gene in the AP2044 strain’s genome, which comes from *Pasteurella multocida via* HGT, may protect ribosomes from macrolides. Under antibiotic selection pressure, random mutations, ARG acquisition *via* HGT, and activation of mobile DNA elements are viable mechanisms (Salehi et al., 2021). Surprisingly, the XDR *A. pittii* AP2044 strain most likely acquired many ARGs *via* HGT, resulting in high levels of resistance to the majority of the antibiotics tested (Table 1). Additionally, a total of four different IS elements next to the ARGs are found in AP2044 plasmids. Several different IS elements were identical (100%) to IS*Alw125*, IS*17*, and IS*Aba2* of *Acinetobacter* spp., which are adjacent to the *aph(3′)-VIa,aph(3″)-Ib*, and *sul2* genes. The *aac(3″)-IIb* gene was located between two IS*30* family elements. As a result, the AP2044 strain’s remarkable XDR capacity was related to the enormous amount of ARGs and IS elements in its genome.

Interestingly, the AP2044 strain had a high level of carbapenem resistance and carried the *bla*_NDM–1_ gene. The presence of NDM, a widespread metallo-β-lactamase (MBL) in *Acinetobacter* was noteworthy (Perez et al., 2007). The increased number and diversity of MBLs in *Acinetobacter* spp. indicated a concerning trend in the global emergence of resistance in this pathogen (Dortet et al., 2014). In *Acinetobacter*, the *bla*_NDM_-type genes were located on either the plasmid or chromosome (Wong et al., 2017). Nevertheless, the AP2044 strain carried two copies of *bla*_*NDM–1*_ genes, which are located on both the plasmid and chromosome. The *bla*_NDM–1_ gene is typically located between two copies of the IS*Aba125* element in NDM-producing *A. baumannii*, forming a composite transposon termed Tn*125* (Bonnin et al., 2012, 2014; Krahn et al., 2016). Except for the insertion of an IS*91* family transposon and deletion of *insE*, the genetic context of *bla*_NDM–1_ on the chromosome was similar to that of Tn*125*. Indeed, by the late 1970s, a worldwide lineage of *A. baumannii* had gained resistance to conventionally known antibiotic families. Further resistance was acquired in transposon lineages in the 1980s as new antibiotics became available (Blackwell et al., 2016). Transposon Tn*125* proved to be the primary vehicle for the spread of *bla*_NDM–1_ in *Acinetobacter* spp. (Poirel et al., 2012). Current observations suggested that the *bla*_NDM–1_ gene originated from an unknown environmental bacterial progenitor species and was integrated into the chromosome of *Acinetobacter* spp. The *bla*_NDM–1_-bearing Tn*125* transposon was most likely derived from such *Acinetobacter* spp. and then transferred to broad-host-range plasmids before being horizontally transferred to *Enterobacteriaceae* and *P. aeruginos*a (Nordmann et al., 2012; Bonnin et al., 2014). Of greater interest was that this NDM-1 producer carried the structure (IS*30*-*bla*_NDM–1_-*ble*_*MBL*_-*trpF*-ORF-Y-family DNA polymerase-*umuD*-ORF-ORF-ORF-IS*Aha3*) surrounding the *bla*_NDM–1_ gene (Figure 1B), which was similar to that found in pAcsw19-2 (Sichuan, Luzhou) (accession no. CP043309.1). These two plasmids originate in the same area, and the personnel mobility is substantial. Moreover, sewage is the origin of pAcsw19-2. Several investigations have suggested that it could be a major source of resistance genes as well as a hotspot for transmitting resistance genes and MGEs to clinical microorganisms. Moreover, the possibility of gene cluster transfer should be considered due to the diversity of the *bla*_NDM–1_ gene environment. In addition, a massive resistance island in the *Acinetobacter*’s genome (Adams et al., 2008) could acquire additional genetic entities for resistance from other bacterial species. Genomic island GI_AP2044-4 (42,734 bp) carried the chromosome-borne *bla*_NDM–1_. Sequence analysis showed that GI_AP2044-4 had 90% query cover and 98.8% sequence similarities with the DNA sequence of *A. pittii* strain ST220 chromosome (accession no. CP029610.1) genome (Supplementary Datasheet 3).

### Identification of the Novel β-Lactamase OXA-1045 and Genetic Environment of *bla*_OXA–1045_

**FIGURE 2:**
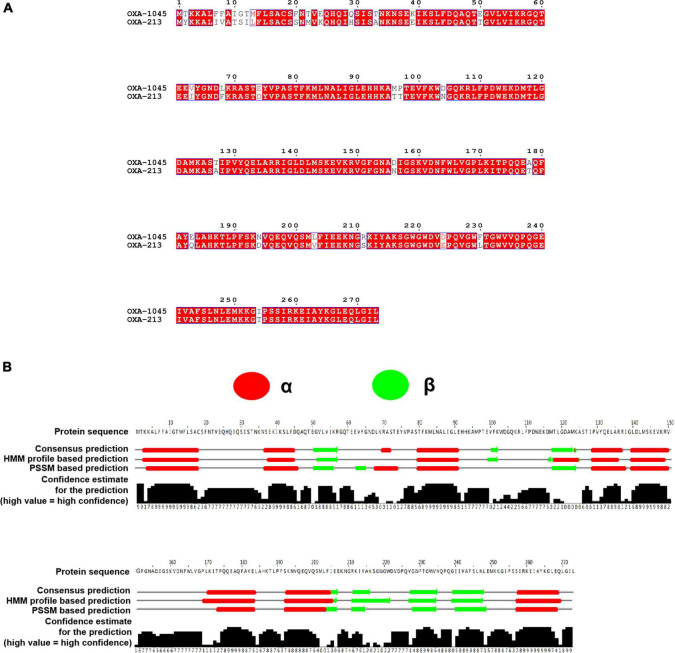
**(A)** Clustal Omega and ESPript 3.0 were used to align the amino acid sequences of OXA-822 and OXA-213. Residues that have been conserved are highlighted in boxes. **(B)** Secondary structure of OXA-1045. The secondary structure was predicted using the neural network-based web service JPred4 with the default settings. Secondary structure elements, α helixes, β sheets.

Previously, two β-lactamase genes located on the chromosome were identified by WGS. One gene encoding an ADC-25-like cephalosporinase and another gene encoding a novel OXA variant were determined to have an 89% aa identity (243/273 aa) and 100% coverage (273/273 aa) compared to OXA-213. The aa sequences of OXA enzymes are quite diverse, and a cutoff of 73.1% of aa identity has recently been proposed as a criterion for dividing OXA subfamilies (Yoon and Jeong, 2021). Therefore, the novel OXA variant belonged to the OXA-213-like subfamily. The Pathogen Detection group at GenBank’s National Center for Biotechnology Information has awarded it the number OXA-1045 (accession no. OL790815) (Evans and Amyes, 2014; Yoon and Jeong, 2021). Sequence alignment of OXA-1045 with OXA-213 revealed 30 aa changes and the secondary structure of OXA-1045 contained nine α helixes and six β sheets (Figure 2). *Acinetobacter* isolates have shown complex interactions with multiple mechanisms of resistance to carbapenems, and the production of naturally occurring OXAs has been the most frequently observed. The predominance of OXAs (OXA-23, OXA-24 or –40, OXA-51, OXA-58, and OXA-143) is the major reason for phenotypic resistance to carbapenems, which have been detected in many parts of the world (Adams-Haduch et al., 2011; Principe et al., 2014; Kamolvit et al., 2015; Labarca et al., 2016). Among these carbapenem-hydrolyzing OXA-type lactamases, *bla*_OXA–23_ is regarded as an intrinsic gene of *Acinetobacter radioresistens*, OXA-51 is intrinsic to *A. baumannii* and the OXA-134 variant is intrinsic to *Acinetobacter schindleri* and *A. lwoffii* (Poirel et al., 2008; Turton et al., 2012; Périchon et al., 2014). OXA-213-like enzymes have been identified to be intrinsic to *A. calcoaceticus* and have been subsequently detected in *A. pittii* (Figueiredo et al., 2012; Tietgen et al., 2021). Phylogenetic analysis of OXA-213-like proteins identified two distinct subgroups within the OXA family. The first group was linked to *A. pittii* and the second group to *A. calcoaceticus* (Supplementary Figure 1).

The genetic environment of *bla*_OXA–1045_ from the AP2044 strain is shown in Figure 1C. The *fxsA* gene, which is situated upstream of the *bla*_*OXA–1045*_ gene, encoded the cytoplasmatic membrane protein. A potential redox protein-coding gene *osmC*-like, a transketolase protein-coding gene *tkt*, and an S adenosylmethionine synthase-coding gene *metK* were positioned upstream of *fxsA*. All *bla*_OXA–1045_ downstream genes were *yncA, ruvC, nnrD*, and *queG*, which encoded the N-acetyltransferase family protein, crossover junction endodeoxyribonuclease, bifunctional NAD(P) H-hydrate repair enzyme, and epoxyqueuosine reductase, respectively. The genetic context of *bla*_OXA–1045_ showed the closest similarity with that of *bla*_OXA–417_ in a BLAST search, which was naturally found on the chromosome of *A. pittii* and belonged to the *bla*_OXA–213–*like*_ family. In addition, there was no mobile element found in the surrounding region of *bla*_OXA–1045_. The above findings implied that the initial location of *bla*_OXA–1045_ was in the chromosome. The presence of an IS upstream of the gene, which acted as a powerful promoter, can boost the production of OXAs (Turton et al., 2006). Hence, there was no evidence of OXA-1045 overproduction.

### Impact of OXA-1045 on Antibiotic Susceptibility to β-Lactams

Among the antibiotics tested, only the MICs of ampicillin, piperacillin, cefazolin, cefoxitin, cefuroxime, ampicillin-sulbactam, and piperacillin-tazobactam for the transformant containing pET28b-OXA1045 (BL21:pET28b-OXA1045) were increased by ≥ 2-fold, as compared to those for the transformant containing pET-28b (BL21:pET28b). In comparison with OXA-213, OXA-1045 elevated the MICs of piperacillin and piperacillin-tazobactam slightly, suggesting that OXA-1045 had a greater impact on piperacillin. Noteworthy, the MICs of ceftazidime, cefotaxime, meropenem, and imipenem for both transformants remained the same as the acceptor strain, demonstrating that the OXA-213 resistance profile to cephalosporins and carbapenem was similar to that of OXA-1045 (Table 4). A previous study has illustrated that the production of all OXAs led to a significant increase in all carbapenem MICs in *A. baumannii*, while no elevation in MICs was observed in *E. coli* (Tietgen et al., 2021). Indeed, we could not rule out the possibility that the effect on MICs is a result of endogenous OXA cooperation with vector-expressed OXA-213-like variants in *Acinetobacter* spp. The results indicated that carbapenemase activity of tested OXAs may be related to the species-dependent effect.

**TABLE 4:** MICs (mg/L) of β-lactams.

	BL21:pET28b_ OXA1045	BL21:pET28b_ OXA213	BL21:pET28b
Ampicillin	4	4	2
Piperacillin	8	4	2
Oxacillin	256	256	256
Cefazolin	2	2	1
Cefoxitin	2	2	0.5
Cefuroxime	2	2	<0.025
Ceftazidime	<0.025	<0.025	<0.025
Cefotaxime	<0.025	<0.025	<0.025
Meropenem	<0.025	<0.025	<0.025
Imipenem	<0.025	<0.025	<0.025
Ampicillin-sulbactam	2/4	2/4	0.5/4
Piperacillin-tazobactam	4/4	2/4	1/4

### Biofilm Production and Detection of Virulence Phenotype

**FIGURE 3:**
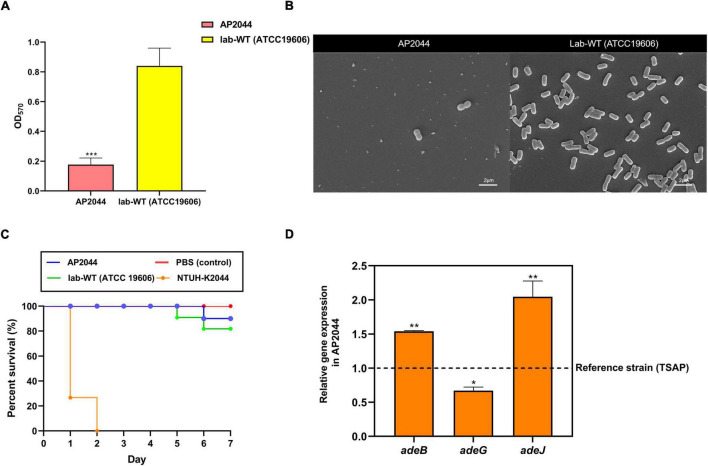
**(A)** Crystal violet quantification of biofilm formation in the AP2044 and Lab-WT strains; Lab-WT was used as positive control and LB broth was used as negative control. **(B)** SEM images of the AP2044 and Lab-WT strains. **(C)** To compare pathogenicity *in vivo*, 15 *Galleria mellonella* larvae were infected with the common strain Lab-WT, typical hypervirulent strain NTUH-K2044, and strain AP2044 under each condition. Death was defined as a lack of reaction or melanization in infected *G. mellonella* at 37°C for 7 days. The mean (*N* = 15 biological replicates) is represented for the data. **(D)** The expression of *adeB*, *adeG*, and *adeJ* genes was quantified *via* qRT-PCR. Gene expression profiles of the strains were normalized to their respective 16S rRNA expression. Data represent the mean (± standard deviation, *SD*; *N* = 4–6 biological replicates). *P < 0.05; **P < 0.01; ***P < 0.001 by Student’s *t*-test against theoretical value.

The biofilm formation capacity was measured in the Lab-WT and AP2044 strains. The OD_570_ values for the Lab-WT and negative control were 0.84 ± 0.12 and 0.14 ± 0.008, respectively. The OD_570_ value for the AP2044 strain was 0.177 ± 0.045, which was a weak biofilm producer. Moreover, the SEM result was consistent with the OD_570_ values obtained by crystal violet staining (Figures 3A,B). The AP2044 strain presented mucoid phenotype, with moist colonies and an elevated surface (Supplementary Figure 2). Mucoid phenotype formation may influence the virulence of pathogenic microorganisms to varying degrees, which has allowed to make significant strides in characterizing the determinants of pathogenic mechanisms in *P. aeruginosa* and *Klebsiella pneumoniae* (Dennis et al., 2018; Ding et al., 2022). A previous study has demonstrated that mucoid *A. baumannii* strains were more virulent than non-mucoid isolates (Shan et al., 2021). Therefore, we analyzed the virulence of AP2044 strain by developing a *Galleria mellonella* infection model. As shown in Figure 3C, such virulence of AP2044 strain was comparable to that of the Lab-WT, which is well known for its lack of virulence (Khalil et al., 2021). The association between the virulence and mucoid phenotype in *Acinetobacter* spp. warrant further investigation. Indeed, the capacity of *A. baumannii* to form biofilm facilitated its survival and persistence in hospital environments (Donlan and Costerton, 2002; Gaddy et al., 2009). This, in turn, contributed to the extensive spread of this pathogen across the globe. Many virulence factors have been implicated in the initial adhesion process of biofilm (Zeighami et al., 2019). Likewise, biofilm development is one of the basic virulence traits of clinical isolates (Khalil et al., 2021). Mahmoud et al. reported biofilm formation as a potent virulence factor in *A. baumanni*, with the strong biofilm producers exhibiting a much greater ability to kill *G. mellonella* larvae than the moderate and weak biofilm producers (Khalil et al., 2021). Accordingly, AP2044 strain was a weak biofilm producer and a low virulence strain. Several previous studies have demonstrated that the majority of XDR and pandrug-resistant (PDR) clinical isolates were weak or non-biofilm producers, which is consistent with the present findings (Qi et al., 2016; Li et al., 2021). There may be a metabolic cost caused by high-level antibiotic resistance, which has been shown to cause a decrease in virulence (Roux et al., 2015). However, the emergence of carbapenem-resistant hypervirulent *A. baumannii* (CR-hvAB) strains presents significant challenges for public health and infection control (Li et al., 2020).

### Relative Gene Expression

Compared to the reference strain, quantitative analysis demonstrated that AP2044 expressed 1. 54-, 0. 67-, and 2.05-fold more *adeB, adeJ*, and *adeG* genes, respectively (Figure 3D). In particular, the transcription level of *adeB* and *adeJ* in AP2044 was significantly overexpressed than that in TSAP (*t*-test, *P* < 0.01). The efflux pump plays a vital role in both biofilm formation and antibiotic resistance, particularly in tigecycline resistance (Lee et al., 2020). The tigecycline is one of the last resort options for XDR strain infection treatment (Wong et al., 2017). *Acinetobacter* has shown superior resistance to almost all available systemic antibiotics and demonstrates an XDR phenotype. Therefore, overcoming antibiotic resistance is the primary challenge of treating *Acinetobacter* infections (Wong et al., 2017). AdeABC in particular has been demonstrated to influence antibiotic sensitivity and to contribute to tigecycline resistance (Ruzin et al., 2007; Roca et al., 2011). The AdeRS two-component system, which consists of a sensor kinase and a response regulator, is in charge of expressing the transcription of the AdeABC efflux pumps. The aa changes or IS element insertion in the AdeRS two-component system can boost the transcription level of AdeABC efflux pumps (Yoon et al., 2013; Lucaßen et al., 2021). AdeIJK is regulated by the TetR-like repressor AdeN, whose overproduction results in antibiotic resistance and contributes to tigecycline resistance. The present findings indicated that AdeABC and AdeIJK overexpression was the cause of tigecycline resistance, which is consistent with previous studies. A previous study have demonstrated that the AdeABC and AdeIJK efflux systems contributed to tigecycline resistance in a synergistic manner (Damier-Piolle et al., 2008).

## Conclusion

In summary, the present study found that XDR *A. pittii* carrying two copies of *bla*_NDM–1_. The *bla*_NDM–1_ was located on the chromosome and plasmid in the *A. pittii* strain, which highlighted the fact that *bla*_NDM–1–_bearing the Tn*125* transposon was most likely a vector of communication between such *Acinetobacter* spp. and uncommon *Enterobacteriaceae* strains. Then, transfer of the antibiotic-resistant plasmid in *Acinetobacter* spp. deserves special attention. The present work also identified a novel OXA variant in the OXA-213 family, OXA-1045, which was able to confer a reduced susceptibility to piperacillin and piperacillin-tazobactam compared to OXA-213. Phenotypic investigations have found that the AP2044 strain was comparable to the wild-type in terms of pathogenicity but with a weaker biofilm structure. In addition, the tigecycline resistance of the AP2044 strain may be due to the overproduction of AdeABC and AdeIJK.

## Data Availability Statement

The datasets presented in this study can be found in online repositories. The names of the repository/repositories and accession number(s) can be found below: NCBI GenBank; CP087716-CP087718; OL790815.

## Author Contributions

JL designed this study. ZD, ZL, and YZ performed the experiments and analyzed the data. ZD and YZ wrote the manuscript. JH and TL uploaded the data and performed analysis of qRT-PCR. YL and ZZ revised the manuscript. All authors contributed to the article and approved the submitted version.

## Conflict of Interest

The authors declare that the research was conducted in the absence of any commercial or financial relationships that could be construed as a potential conflict of interest.

## Publisher’s Note

All claims expressed in this article are solely those of the authors and do not necessarily represent those of their affiliated organizations, or those of the publisher, the editors and the reviewers. Any product that may be evaluated in this article, or claim that may be made by its manufacturer, is not guaranteed or endorsed by the publisher.

## Footnotes

1
https://cge.cbs.dtu.dk/services/ResFinder-3.2/

2
https://blast.ncbi.nlm.nih.gov/Blast.cgi

3
https://www.biologicscorp.com/tools/GCContent/#.XnIhjqgzZPY

4
https://isfinder.biotoul.fr/blast.php

5
http://www.mgc.ac.cn/VFs/main.htm

6
https://espript.ibcp.fr/ESPript/ESPript/

7
https://www.compbio.dundee.ac.uk/jpred/

8
http://bldb.eu/BLDB.php?class=D#OXA

9
https://cge.cbs.dtu.dk/services/
