# Supplementary material for: Phenotypic and Genotypic Characteristics of a Tigecycline-Resistant Acinetobacter pittii Isolate Carrying blaNDM–1 and the Novel blaOXA Allelic Variant blaOXA–1045
Source: Front Microbiol. 2022 May 4;13:868152. doi: 10.3389/fmicb.2022.868152 (PMC9116503; doi:10.3389/fmicb.2022.868152)
Supplement: Supplementary file 1 [file Data_Sheet_1.docx]

Supplementary Material

**Table S1 Primers used in this study**

| Gene | Primer | Sequence (5ʹ-3ʹ) | Product size | Purpose | Reference |
| --- | --- | --- | --- | --- | --- |
| *16s* rRNA | F | AGAGTTTGATCCTGGCTCAG | 1465 | Screen | This study |
|  | R | CGGTTACCTTGTTACGACTTC |  |  |  |
| *adeB* | F | GAATAAGGCACCGCAACAAT | 124 | Screen | (Nowak et al., 2015) |
|  | R | TTTCGCAATCAGTTGTTCCA |  |  |  |
| *adeG* | F | TGAACGATGCTGCTCAAAAC | 681 | Screen | (Nowak et al., 2015) |
|  | R | CTCCAGCTGTCAACCAGACA |  |  |  |
| *adeJ* | F | CTTGGTGTAACTGCCGGATT | 605 | Screen | (Nowak et al., 2015) |
|  | R | TGAGCACCAGACTCACGTTC |  |  |  |
| *adeB* q-PCR | F  R | TGCTACAACGGATTCTTCAGGAACC | 146 | Quantitative real-time PCR | This study |
|  | R | CTAAGGCTTGTTGGCGGACGAC |  |  |  |
| *adeG* q-PCR | F | GCCTAAGGCGCCTCGGTCTT | 127 | Quantitative real-time PCR | (Yang et al., 2019) |
|  | R | GGAGCGCTCAACCAGAAATATT |  |  |  |
| *adeJ* q-PCR | F | GCGGGTATTCTCACGCTAAC | 110 | Quantitative real-time PCR | (Yang et al., 2019) |
|  | R | TTCAACTGTTTCAGCCGATG |  |  |  |
| *16s* q-PCR | F | CAGCTCGTGTCGTGAGATGT | 150 | Quantitative real-time PCR | (Lin et al., 2017) |
|  | R | CGTAAGGGCCATGATGACTT |  |  |  |
| PET28AVF2AVF2 | F | CGGATATAGTTCCTCCTTTC | 6135 | Sequence | This study |
| PET-VF | R | CCCGCGAAATTAATACGACTCAC |  |  |  |
| OXA-1045 | F | tgactggtggacagcaaatgggtcgggatccgATGACTAAAAAAGCTCTT | 835 | Restructure | This study |
|  | R | gatctcagtggtggtggtggtggtgctcgagTTATAAAATACCTAGCTGC |  |  |  |
| OXA-213 | F | tgactggtggacagcaaatgggtcgGGATCCGATGTACAAAAAAGC | 835 | Restructure | This study |
|  | R | gatctcagtggtggtggtggtggtgCTCGAGTTATAAGATACCCAG |  |  |  |

Note: The sites for homologous regions are underlined.

**Table S2 Resistance Gene Distribution in *Acinetobacter baumannii* strain ATCC19606**

| Resistance Gene | Identity % | Query/Template Length | Position in Context | Predicted Phenotype | Accession number |
| --- | --- | --- | --- | --- | --- |
| *bla*_ADC-25_ | 97.14 | 1152 / 1152 | 1125291..1126442 | Beta-lactam resistance | [EF016355](http://www.ncbi.nlm.nih.gov/nuccore/EF016355) |
| *bla*_OXA-98_ | 99.88 | 825 / 825 | 2110399..2111223 | Beta-lactam resistance | [EU255288](http://www.ncbi.nlm.nih.gov/nuccore/EU255288) |
| *sul2* | 100 | 816 / 816 | 3895743..3896558 | Sulphonamide resistance | [AY034138](http://www.ncbi.nlm.nih.gov/nuccore/AY034138) |

**Table S3 Overall Features of the *Acinetobacter pittii* Strain AP2044 Genomic Islands**

| **GIs_id** | **Location (Start-End)** | **Length (bp)** | **G+C%** | **Closest Match in Genbank (Query Cover and Identity)** | **Resistance Genes and Mobile Genes Carried** |
| --- | --- | --- | --- | --- | --- |
| AP2044-1 | 3798534-3805118 | 6584 | 28.7 | *Acinetobacter pittii* strain 2014N05-125 chromosome, complete genome (57%, 97.3%) | IS*Aba21* |
| AP2044-2 | 2722732-2744500 | 21768 | 33.7 | *Acinetobacter baumannii* strain B8300 chromosome, complete genome (76%,99.3%) | IS*3* famliy transposase |
| AP2044-3 | 1435513-1441067 | 5554 | 35.8 | *Acinetobacter pittii* strain ST220 chromosome, complete genome (73%, 99.9%) | IS*3* famliy transposase |
| AP2044-4 | 3146433-3189167 | 42734 | 42.8 | *Acinetobacter pittii* strain ST220 chromosome, complete genome (90%, 98.8%) | IS*91* family transposase, NDM-1, two IS*Aba125*, Tn*As3* transposon |
| AP2044-5 | 2470908-2485821 | 14913 | 35.9 | *Acinetobacter pittii* strain ST220 chromosome, complete genome (89%, 99.8%) | IS*3* famliy transposase |
| AP2044-6 | 2435308-2447294 | 11986 | 31.8 | *Acinetobacter seifertii* strain AS6 chromosome, complete genome (68%,90.8%) | IS*4* famliy transposase, IS*Aba22* |

**Reference：**

Lin, F., Xu, Y., Chang, Y., Liu, C., Jia, X., and Ling, B. (2017). Molecular Characterization of Reduced Susceptibility to Biocides in Clinical Isolates of Acinetobacter baumannii. *Front Microbiol* 8**,** 1836. doi: 10.3389/fmicb.2017.01836.

Nowak, J., Seifert, H., and Higgins, P.G. (2015). Prevalence of eight resistance-nodulation-division efflux pump genes in epidemiologically characterized Acinetobacter baumannii of worldwide origin. *J Med Microbiol* 64(6)**,** 630-635. doi: 10.1099/jmm.0.000069.

Yang, Y.S., Chen, H.Y., Hsu, W.J., Chou, Y.C., Perng, C.L., Shang, H.S., et al. (2019). Overexpression of AdeABC efflux pump associated with tigecycline resistance in clinical Acinetobacter nosocomialis isolates. *Clin Microbiol Infect* 25(4)**,** 512.e511-512.e516. doi: 10.1016/j.cmi.2018.06.012.

**Figure S1**

**
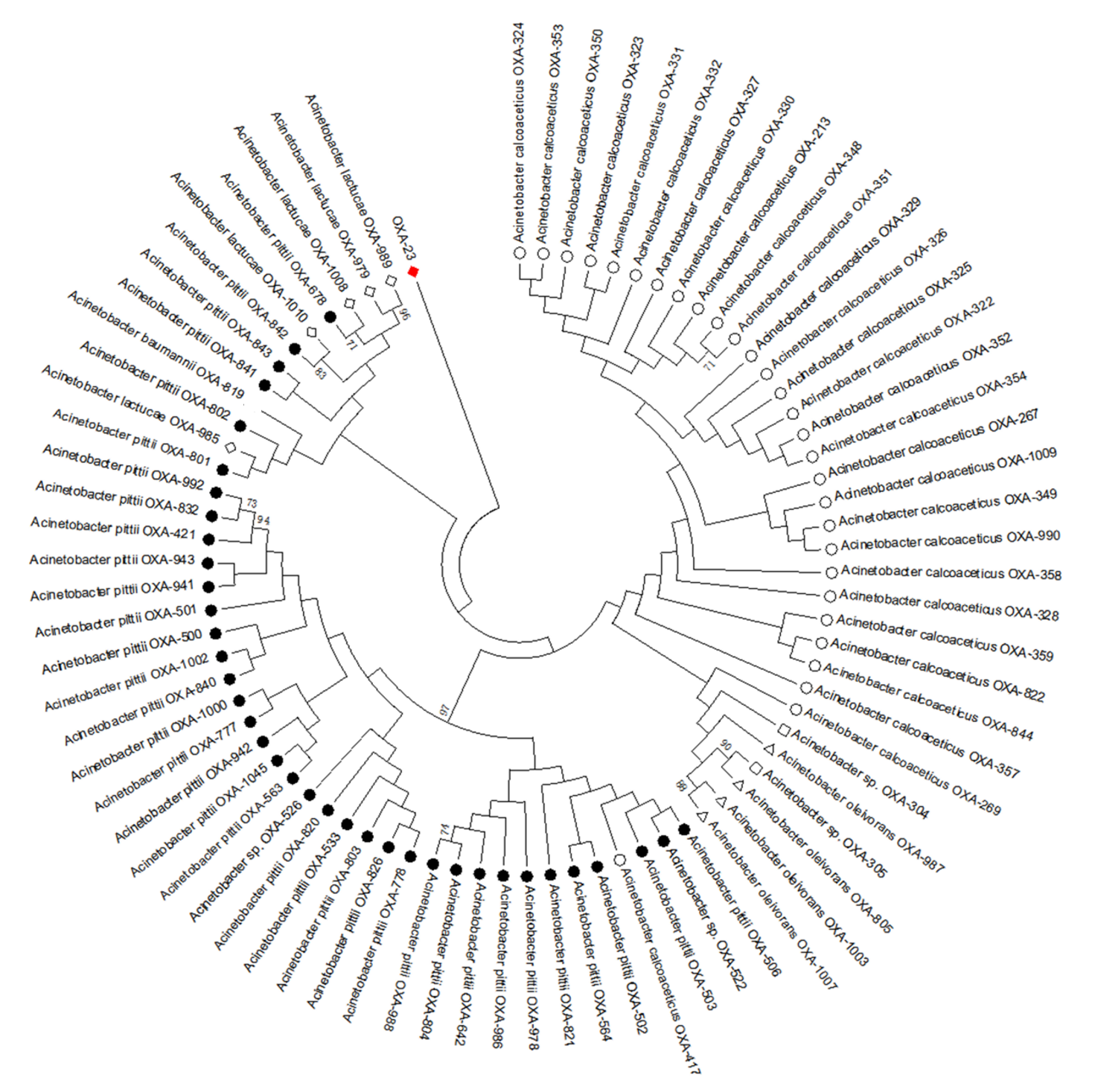
**

Maximum Likelihood method based on an alignment of 78 OXA-213-like β-lactamase amino acid sequences with OXA-23 as the out-group. The maximum-likelihood tree was calculated using MEGA 7.0 (bootstrapping, n=500); only bootstrapping values >70 is shown. represented *A.calcoaceticus* group*,* represented  *A.pittii* group*,* and represnted out-group (OXA-23).

**Figure S2**

**
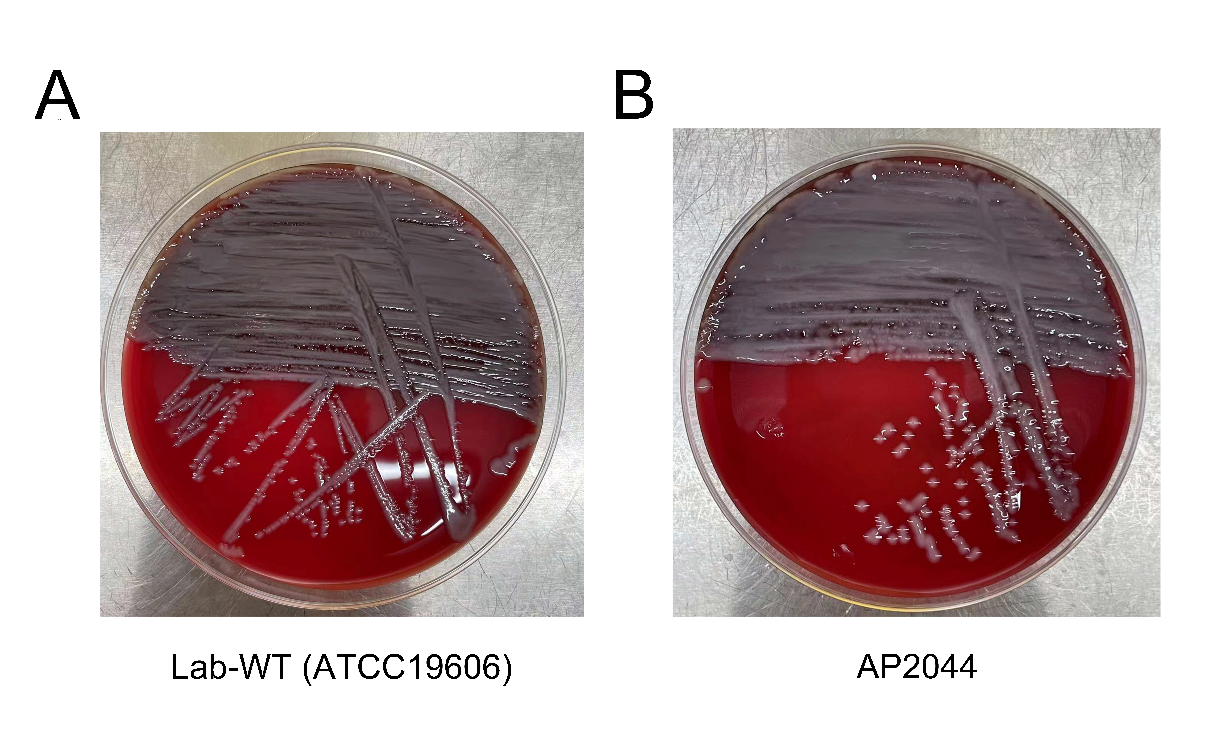
**

Non-mucoid *A. baumannii* Lab-WT (ATCC19606) and mucoid *A. pittii* (AP2044) were passaged on blood agar and in broth.
